# Imaging panorama in postoperative complications after liver transplantation

**DOI:** 10.1093/gastro/gov057

**Published:** 2015-11-02

**Authors:** Binit Sureka, Kalpana Bansal, S Rajesh, Amar Mukund, Viniyendra Pamecha, Ankur Arora

**Affiliations:** ^1^Department of Radiology/Interventional Radiology, Institute of Liver and Biliary Sciences, Vasant Kunj, New Delhi, India; ^2^Department of Hepatobiliary Surgery, Institute of Liver and Biliary Sciences, Vasant Kunj, New Delhi, India

**Keywords:** liver transplant, postoperative complications, imaging

## Abstract

The liver is the second most-often transplanted solid organ after the kidney, so it is clear that liver disease is a common and serious problem around the globe. With the advancements in surgical, oncological and imaging techniques, orthotopic liver transplantation has become the first-line treatment for many patients with end-stage liver disease. Ultrasound, and Doppler are the most economical and cost-effective imaging modalities for evaluating postoperative fluid collections and vascular complications. Computed tomography (CT) is used to confirm the findings of ultrasound and look for pulmonary complications. Magnetic resonance imaging (MRI) is used for the diagnosis of biliary complications, bile leaks and neurological complications. This article illustrates the imaging options for diagnosing the various complications that can be encountered in the postoperative period after liver transplantation.

## Introduction

Cirrhosis is a chronic liver disorder and is caused by a variety of diseases and is associated with a significant increase in mortality and many more morbidities. These diseases cause progressive liver damage and ultimately liver failure and death. The disease burden and the healthcare costs are expected to rise over the next 20 years, given that the percentage of patients with Hepatitis C virus (HCV)-related cirrhosis is predicted to almost double [1]. According to the Global Burden of Disease study conducted by the World Health Organization, liver cancer caused 325 815 disability-adjusted life years (DALYs), and cirrhosis of the liver caused 206 917 DALYs in 2012 [2]. Liver transplantation is the only treatment option available for end-stage liver disease with excellent survival rates.

Liver transplantation can be performed using both cadaveric and living donor transplantations, but an overall higher cost has been reported with living donor transplantations. The overall economic burden of cost for liver transplantation includes direct costs (drug and hospitalization costs) and indirect costs (due to loss of work productivity and reduction in health-related quality of life). This overall cost should also include the post-surgical management of complications, which should be taken into account when determining a patient’s fitness and the cost-effectiveness of liver transplantation. These complications are unpredictable but are common and expensive. The irony of the situation is that these incremental costs due to liver transplantation complications are both unknown and towering for both the hospital and the payor, but the hospital’s profits are not affected substantially and the payor bears the financial burden [3, 4].

The early detection and treatment of postoperative complications (within one year) have contributed significantly to reducing morbidity, mortality and healthcare costs and ultimately improved graft and patient survival. Imaging plays a crucial role in detecting the complications that can lead to graft failure. While imaging is not used to diagnose allograft rejection, it plays an important role for identifying complications that can mimic rejection. Various complications that can be seen are vascular, biliary, fluid collections, gastrointestinal, pulmonary, neurological, neoplasms and rejection (Table 1).

**Table 1.** Postoperative complications after liver transplantation

**Vascular complications**	**Hepatic artery complications** Hepatic arterial thrombosis (HAT) Hepatic arterial stenosis (HAS) Hepatic artery pseudoaneurysm Arterioportal fistula and celiac artery stenosis **Portal vein complications** Portal vein thrombosis (PVT) Portal vein stenosis **Hepatic veins and inferior vena cava complications** Thrombosis Stenosis S**plenic artery steal syndrome (SASS)** **Liver ischemia/infarction**
**Biliary complications**	Stricture, fistula, sphincter of Oddi dysfunction Stone/cast/sludge, biliary cast syndrome Bile leak Ductal ischemia Recurrent biliary disease Mucocele of cystic duct remnant
**Fluid collections**	Seroma Hematoma Abscess
**Abdominal complications**	Bleeding Bowel obstruction Internal and external hernias Perforation Pneumatosis intestinalis Mesenteric ischemia Stercoral and infective colitis Malposition of catheters
**Pulmonary complications**	Pleural effusion Atelectasis Pneumonia (bacterial, viral and fungal) Pulmonary edema Acute respiratory distress syndrome (ARDS) Alveolar hemorrhage Malposition of tubes and catheters
**Neurological Complications**	Hepatic encephalopathy Cerebral edema Central nervous system infections Central pontine and extrapontine myelinolysis Acquired hepatocerebral degeneration Seizures Posterior reversible encephalopathy syndrome
**Neoplasms**	
**Rejection**	

## Surgical technique

The standard whole-liver transplantation includes four vascular anastomoses (portal vein, hepatic artery, suprahepatic and infrahepatic inferior vena cava (IVC) and one biliary anastomosis. In conventional arterial anatomy, the hepatic artery anastomosis is an end-to-end anastomosis between the donor common hepatic-splenic artery branch point or celiac axis with an aortic Carrel patch and the recipient right and left hepatic artery bifurcation or gastroduodenal-proper hepatic artery bifurcation [5]. A portal vein anastomosis is usually an end-to-end anastomosis between the two portal veins. In cases with portal vein thrombosis, adequate portal inflow can be established using an interposition or venous jump graft from the superior mesenteric vein or splenic vein to donor portal vein [6]. In the conventional bicaval technique, the retrohepatic IVC of the recipient is usually resected, and the IVCs of the recipient and the donor are sutured with an end-to-end anastomosis between the superior and inferior ends. The piggyback technique is the standard technique presently used in most institutions. An end-to-side anastomosis is made between the donor IVC and the common stump of the recipient hepatic veins [7]. Biliary reconstruction during liver transplantation is accomplished using either a choledochocholedochostomy or a Roux-en-Y choledochojejunostomy [8].

## Vascular complications

Vascular complications are suspected in cases of post-transplant liver failure, bile leakage, gastrointestinal, abdominal, or biliary bleeding and sepsis. The incidence of vascular disorders is approximately 9% and is usually evident in the early postoperative period.

### Normal Doppler and Variability in the Postoperative Period

Doppler examination of the liver transplant involves evaluation of the main hepatic artery (at the porta hepatis, anastomotic site and its intrahepatic branches), main portal vein and its branches, hepatic veins and the IVC. The normal acceleration time has a rapid systolic upstroke with continuous diastolic flow. The mean peak systolic velocity (PSV) is around 103 cm/s, acceleration time is < 0.08 seconds, and the resistive index (RI) is between 0.5 and 0.8. Transient increased RI (RI > 0.8, absent or even reversed diastolic flow) may be seen in almost 50% of patients in the immediate postoperative period of < 72 hours. The RI usually normalizes within two weeks and is not associated with graft rejection. This transient elevation of RI may be due to allograft edema, increased cold ischemia time, increased portal flow or vessel spasm. Decreased hepatic arterial RI (RI < 0.5) is a more unpropitious finding than increased RI and is usually of concern for arterial complications. Decreased RI is usually due to anastomotic site edema and resolves in a few days [9, 10]. Complications involving the hepatic artery are hepatic artery stenosis, thrombosis, pseudoaneurysm, arteriovenous fistula and, rarely, celiac stenosis.

### Hepatic artery thrombosis

Hepatic artery thrombosis (HAT) is the most common complication of orthotopic liver transplantation and is seen in the first two weeks post surgery. Its incidence varies from 4–12% in adults and is ∼42% in children. HAT is generally broken down into two types depending on the time frame during which the vascular flow occlusion occurs: early HAT, (within the first month post liver transplant) and late thrombosis HAT (after the first postoperative month. Risk factors predisposing to HAT are prolonged cold ischemia time of the graft, significant difference in caliber between the donor and recipient hepatic arteries (especially in children), interpositional conduit for the anastomosis, previous stenosis in the celiac axis, ABO incompatibility, technical errors, cytomegalovirus infection and acute rejection. Diagnosis of HAT is established by the absence of flow in the hepatic artery proper and in the intrahepatic arteries on color Doppler sonography. Impending thrombosis is seen as loss of diastolic flow being the first change, followed by a diminished systolic peak and finally no color flow in the hepatic artery. Clinically, there may be fever, biliary leak, liver abscess, alteration in the liver function tests and raised lactate-pyruvate levels. Doppler ultrasound can detect HAT in the pre-symptomatic stage, allowing early reperfusion.

**Figure 1.**
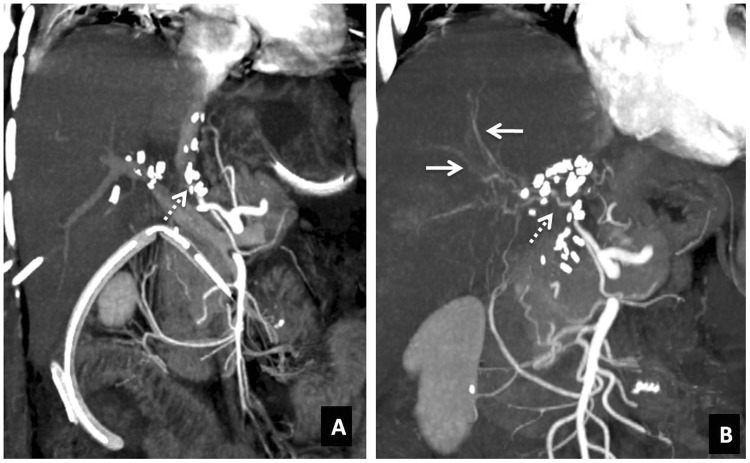
Hepatic artery thrombosis. (A) Coronal-oblique MIP contrast-enhanced CT images acquired in the arterial phase demonstrating abrupt termination of the hepatic artery (HA) at the anastomotic site (interrupted arrow) with non-visualization of the intrahepatic branches of HA. (B) Post-thrombolysis image showing opacification of the segment of the HA beyond the anastomosis (interrupted arrow) as well as the intrahepatic arteries (solid arrows).

It is essential to evaluate the hepatic artery at different sites as normal hepatic flow detected at the porta hepatis does not exclude HAT at a different location, especially in cases with recent alteration of liver function tests, bile leaks with intrahepatic biloma formation, anastomotic bile duct stricture, perihepatic fluid collections, biliary peritonitis and sepsis. False-positive results on Doppler examination are seen in patients with low cardiac output, arterial spasm or severe parenchymal edema, whereas false-negative results may occur as a result of periportal collateralization subsequent to chronic thrombosis. Collateral channels show extra- or intrahepatic parvus tardus waveform, acceleration time > 0.08 second and RI < 0.5. The tardus-parvus waveform in intrahepatic branches does not exclude HAT but rather indicates the presence of either upstream stenosis or thrombosis. In suspicious cases, computed tomography angiography (CTA) or magnetic resonance angiography (MRA) should be performed. On angiography, thrombus appears as a filling defect within the hepatic artery or as amputated hepatic artery (Figure 1). Secondary signs such as intrahepatic infarction areas, bilomas, abscesses and biliary strictures may be also noted [9, 10]. Memeo *et al*. recommended systematic CTA at the end of the first postoperative week to detect significant hepatic artery stenosis (> 50%) and false aneurysms to decrease the incidence of late onset HAT [11, 12].

### Hepatic artery stenosis

Hepatic artery stenosis (HAS) is the second most common vascular complication reported in 2–10% of transplant recipients. This complication occurs at the anastomotic site, usually within three months of transplantation. Risk factors for HAS are clamp injury, intimal trauma from a perfusion catheter and disruption of the vasa vasorum. On Doppler evaluation, the stenotic segment shows turbulence and aliasing with PSV > 200 cm/s. A tardus parvus waveform maybe seen distally. False-positive cases may be seen in presence of vascular kinks and anastomotic site edema. Correction of the Doppler angle can help differentiate this from true stenosis [13, 14]. It has recently been suggested that the combination of the tardus parvus pattern and an optimal PSV cutoff ≤ 48 cm/s greatly improved the positive predictive value and reduced the false-positive rate in the diagnosis of HAS [15]. Stenosis of the recipient celiac axis can cause decreased hepatic arterial resistive indices in the transplanted liver, mimicking hepatic artery stenosis.

Platt *et al*. in their study of 46 patients concluded that abnormal values for both RI and acceleration time were 67% sensitive and 96% specific for stenosis [13]. When at least one abnormal value was found on Doppler imaging, sensitivity and specificity for stenosis were 81% and 60%, respectively. Digital subtraction angiography (DSA) is the gold standard for diagnosis of vascular complications and allows a concomitant intervention procedure. Vogl *et al*. compared DSA and CT in the detection of HAT in 24 liver-transplant recipients and found CT to be 89% sensitive and 100% specific [16]. Legmann *et al*. found a sensitivity of 100% for CTA with maximum intensity projection in the detection of HAT [17]. Volume rendering (VR) technique in CTA is a more accurate, better and useful noninvasive technique for detecting vascular complications in liver transplant patients than maximum intensity projection (MIP) and shaded surface display techniques. These techniques use < 10% of the image data, while VR displays nearly all of the volume of data [18]. This allows VR three-dimensional CTA to display multiple overlapping vessels by providing vessel depth similar to DSA.

Recently, contrast-enhanced ultrasound (CEUS) has begun providing real-time angiography-like images of vessels and allowing the accurate diagnosis of arterial diseases such as HAT. Zheng *et al*. in their series of 47 liver transplant recipients who underwent CEUS found an accuracy of 91.5%, sensitivity of 92.3%, specificity of 87.5%, positive predictive value of 97.3% and a negative predictive value of 70% [19]. CEUS corrected false-positive findings on color Doppler ultrasound in seven of 47 cases. They concluded that CEUS is a useful noninvasive technique for detecting HAS in liver transplant patients because it provides comprehensive information including the presence, location, degree and type. A positive CEUS finding would suggest DSA as the next step rather than a CT scan and may thereby alter the clinical imaging algorithm. Compared with CTA and MRA, CEUS has the following advantages. (i) The ultrasound contrast agents used in CEUS are not nephrotoxic, and adverse reactions are very rare because the gas within microbubbles is eliminated from circulation by exhalation via the lungs; this is particularly important for transplant recipients as many have renal insufficiency. (ii) Patients with severe HAS on CEUS could directly undergo interventional angiography, and this may save critical time. (iii) CEUS is easily carried out as a bedside procedure for patients in the intensive care unit, and (iv) it lacks radiation and can be used as a repeated follow-up modality for vessel assessment.

### Hepatic artery pseudoaneurysm

Hepatic artery pseudoaneurysm is rare and results from infection or iatrogenic event secondary to a biopsy or angioplasty. Clinically the patient may present with features of shock, hemobilia or upper gastrointestinal bleeding. On ultrasound, a cystic structure may be seen in the periportal region along the course of the hepatic artery, which on Doppler evaluation reveals turbulent arterial flow with an internal ying-yang phenomenon.

### Portal vein complications

**Figure 2.**
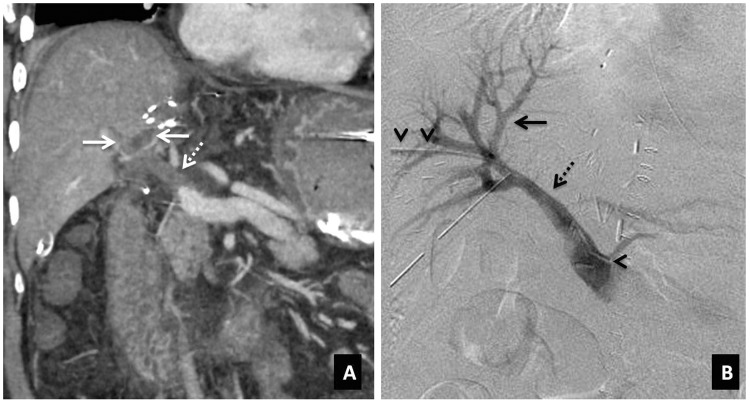
Portal vein thrombosis. (**A**) Coronal-oblique MIP image demonstrating thrombosis of the extrahepatic portal vein (interrupted arrow) as well as its intrahepatic branches (solid arrows). (**B**) Post-thrombolysis DSA image showing recanalization of the main portal vein (interrupted arrow) and the intrahepatic portal venous radicals (solid arrow). Arrowheads denote the percutaneously placed angiographic catheter with its tip at the splenoportal confluence.

**Figure 3.**
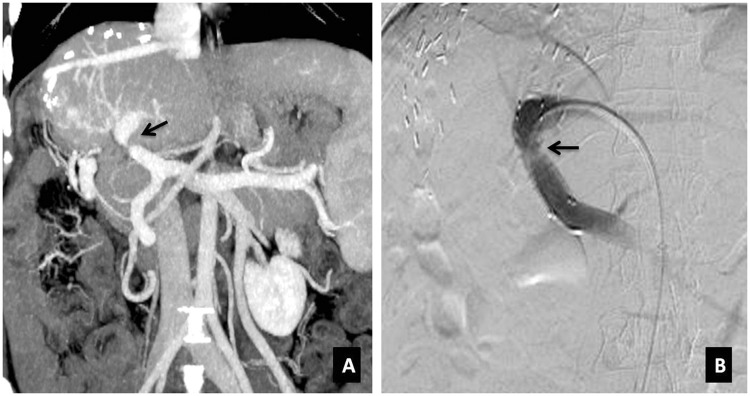
Portal vein stenosis. (**A**) Coronal-oblique MIP image showing short-segment stenosis of the portal vein (arrow) at the anastomotic site. (**B**) Post-angioplasty and stenting DSA image demonstrating recanalization of the vein (arrow).

Post transplant, the normal portal vein has an antegrade, continuous hepatopetal monophasic flow with respirophasicity. The portal venous velocity is highly variable and shows turbulent flow that tends to decrease on serial examinations. The incidence of portal vein thrombosis (PVT), and stenosis is ∼1–2%. PVT is usually seen within one month, and portal vein stenosis is a late complication (after six months) of liver transplant. The risk factors are vessel misalignment, differences in the caliber between donor and recipient vessels, stretching of the portal vein anastomosis, previous portal vein surgery or thrombosis in the recipient portal vein, decreased portal inflow, increased resistance from suprahepatic kinking of IVC and hypercoagulable states. Clinically, the patient presents with signs of portal hypertension, hepatic failure, massive ascites or edema. On ultrasound, an echogenic thrombus with no color flow in the portal vein is seen. On contrast-enhanced CT, non-opacification of the portal vein is seen (Figure 2). Occasionally, portal cavernoma may form with chronic PVT. Portal vein stenosis is more common in pediatric liver transplants and is diagnosed when the PSV at the anastomotic site is > 125 cm/s or the anastomotic-to-preanastomotic velocity ratio is 3 : 1. CT and MR angiography are confirmatory (Figure 3). On portography, stenosis is considered hemodynamically significant when a pre-stenotic/post-stenotic pressure gradient is > 5 mmHg [10, 20, 21].

### Inferior vena cava and hepatic vein complications

Thrombosis and stenosis of hepatic veins and IVC are late (usually six months) but rare complications (< 1%) after transplant. Risk factors are size discrepancy between the donor and recipient vessels, suprahepatic IVC kinking from organ rotation, fibrosis, chronic thrombus, neo-intimal hyperplasia, hypercoagulability, compression from graft edema and adjacent fluid collections as well as transplants in pediatric patients. A piggyback anastomosis is more prone to bleed due to cavocaval dehiscence. Clinical manifestations may vary from lower extremity edema to Budd-Chiari syndrome (congested tender hepatomegaly, ascites, pleural effusion).

Normal Doppler waveforms in hepatic veins and IVC show a triphasic pattern. Stenosis is suspected when there is a clinical suspicion along with any of these findings on Doppler ultrasound: turbulent flow, color aliasing, increased PSV at the stenotic segment, pre-/post-anastomotic velocity ratio > 3:1, monophasic waveform with pulsatility index (PI) < 0.45, reversal of hepatic venous flow and indirect signs such as reduced caliber and prestenotic dilatation of the hepatic veins. Biphasic or triphasic hepatic venous waveform excludes significant stenosis. Thrombus is seen as echogenic material within the vessel lumen with absence of color flow on Doppler [10, 20, 21].

### Splenic artery steal syndrome

Splenic artery steal syndrome (SASS) is seen in the immediate postoperative period and is a rare entity. However, in patients with pre-existing portal hypertension and splenomegaly, this can be an overlooked cause of graft ischemia following liver transplantation. The most objective angiographic definition was provided by Uflacker *et al*.,** who only diagnosed splenic steal syndrome when there was visualization of the hepatic artery during the portal-venous phase of the angiogram [22]. On Doppler evaluation, increased RI is seen in intra- and extrahepatic arteries accompanied by increase in PSV in portal and splenic veins. On angiography, a splenic artery diameter > 4 mm or 150% of the hepatic artery, enlarged gastroduodenal artery (GDA) and sluggish flow in the hepatic arteries in an appropriate clinical setting may suggest the diagnosis [23–25]. Cases of GDA steal have also been mentioned in the literature [26]. There is no consensus treatment guideline for splenic artery steal syndrome; however, recent studies have demonstrated coil embolization of the splenic artery to be safe and effective [27, 28].

### Liver ischemia/infarction

Most cases of liver ischemia and infarction are caused by hepatic arterial complications. Ischemia appears as hypo-attenuating areas within the liver parenchyma. The differential diagnosis of hypo-attenuating areas in the transplanted liver includes rejection, ischemia, hepatitis and cholangitis. Infarcts can have a variable sonographic appearance with clear or ill-defined margins with central hypoechoic areas due to liquefaction and necrosis. Superimposed infection with abscess formation may show intraparenchymal gas within the infarct. On CT, infarcts are typically wedge-shaped, low-attenuation peripheral lesions having a territorial distribution [10, 29].

## Biliary complications

Biliary complications occur in 6–34% of liver transplants. It is the second most common cause of liver dysfunction, exceeded only by rejection. Complications include leaks, strictures, stones or sludge, dysfunction of the sphincter of Oddi and recurrent biliary disease.

**Figure 4.**
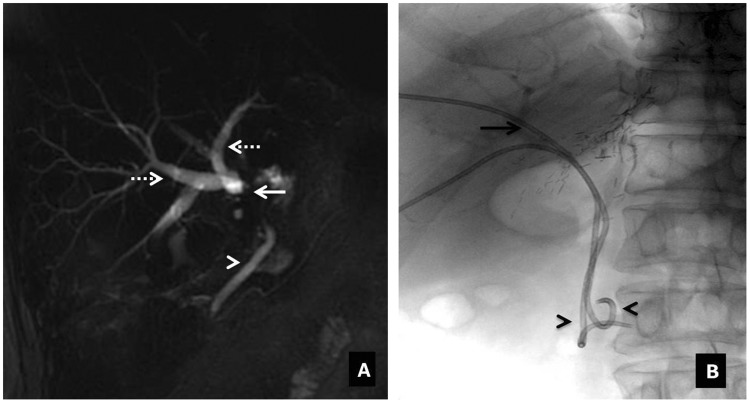
Anastomotic biliary stricture. (**A**) Coronal thick-slab three-dimensional MRCP image demonstrating a stricture at the choledocho-choledochal anastomotic site (solid arrow) with resultant moderate upstream dilatation of the intrahepatic biliary radicals (interrupted arrows). The common bile duct (arrowhead) is also mildly dilated. (**B**) Post-percutaneous transhepatic biliary drainage (PTBD) fluoroscopic image showing drainage catheters in the anterior and posterior sectoral ducts (arrow) with their tips in the second part of duodenum (arrowheads).

Strictures can be at the anastomotic or non-anastomotic site. Stricture at the anastomotic site is usually caused by fibrotic proliferation or, less commonly, by ischemia. Non-anastomotic strictures can occur as a consequence of HAT or without HAT. Non-anastomotic strictures without HAT are separately grouped into ischemic type biliary lesions and are thought to be as a result of perioperative ischemia, chronic disturbance in the bile flow, inflammation and fibrous remodelling. The risk factors for non-anastomotic strictures are ischemia, infection, recurrent biliary disease, micro-angiopathic injury (prolonged warm or cold ischemia times), immunogenic injury (ABO incompatibility, chronic ductopenic rejection, primitive sclerosing cholangitis) and cytotoxic injury by bile salts. The last three factors are associated with ischemic-type biliary lesions. Correlation with imaging and laboratory parameters is mandated to diagnose obstruction as mild dilatation of the biliary tree can be observed in the postoperative period in the absence of obstruction. Diffuse dilatation of the biliary tree may be due to papillary dyskinesia (sphincter of Oddi dysfunction) as a result of devascularization/denervation of the papilla of Vater in the intraoperative period [30, 31]. Two patterns of non-anastomotic strictures are seen: (i) strictures occurring at the hepatic hilum and extending peripherally, and (ii) multiple intrahepatic biliary strictures as the vascularity of the proximal and of the intrahepatic ducts are solely derived from the reconstructed hepatic artery. On ultrasound or magnetic resonance cholangiopancreatography (MRCP), dilated intrahepatic ducts and proximal common bile duct to the level of the strictured segment is seen (Figure 4). If there is biliary dilatation, careful assessment of the hepatic artery should be performed to look for stenosis or thrombosis. False-positive diagnosis of stricture can be made when there is donor-to-recipient common bile duct disproportion and redundancy of the common duct with kinking. In such cases, comparison with intra-operative cholangiogram may be helpful to confirm stability.

Boraschi *et al*. found that MRCP is a reliable technique for detecting biliary complications post liver transplant. They recommend using endoscopic procedure on patients for whom therapeutic procedures are advocated or whose MRCP results are equivocal [32]. The sensitivity, specificity, positive predictive value and negative predictive value of the reviewers for the detection of all types of biliary complications in patients with orthotopic liver transplantation were 98%, 94%, 94%, and 98%, respectively in their study. They concluded that MRCP is a reliable technique for detecting post-orthotopic liver transplantation biliary complications and should be recommended before planning therapeutic interventions.

Biliary leaks are suspected when there is recent development of free fluid or intra / perihepatic fluid collection (biloma). Bile leaks usually occur within the first three postoperative months. The most common site of leak is the anastomotic site (technical) or the T-tube entry point. Non-anastomotic leaks are associated with hepatic artery thrombosis in the majority of cases, followed by immunologic and cytotoxic injury induced by bile salts. Cholangiography is diagnostic. T1-weighted MRCP using hepatospecific contrast agents may be used as the contrast agent gets excreted within bilomas or perihepatic free fluid [21, 33–35].

Stones, casts and sludge are rare but important causes of biliary obstruction. Casts and sludge are seen within one year, whereas stones are usually seen after one year of transplant surgery. Risk factors identified are bile stasis due to biliary strictures, hepaticojejunostomy site, T-tube/stent placement, ischemia, infections and alteration in bile composition. Biliary cast syndrome (BCS) refers to the presence of hard, dark lithogenic material within the biliary system, and its incidence is reported to be between 4–18% in the literature. Significant risk factors associated with BCS are longer warm ischemic time, hepatic artery stenosis, strictures, renal replacement and cyclosporine therapy. On MRCP, biliary casts, sludge or stones appear as hypointense filling defects surrounded by a thin rim of hyperintense bile. Stones are differentiated from casts and sludge by their smooth margins and rounded configuration [36–38].

Mucocele of the cystic duct remnant is a rare complication resulting from ligation of the cystic duct both proximally and distally. It is seen as a round fluid collection compressing and causing obstruction of the common bile duct [29]. Ductal ischemia is associated with complications such as bile leak (fistula), ductal scarring with fibrosis (stenosis) and bile collection (biloma). Recurrent biliary disease is suspected in patients who have undergone transplantation for end-stage primary sclerosing cholangitis on whom imaging demonstrates ductal dilatation, irregularity with skip areas, diverticulum-like outpouchings and wall thickening in the setting of a normal Doppler arterial waveform.

## Fluid collections

**Figure 5.**
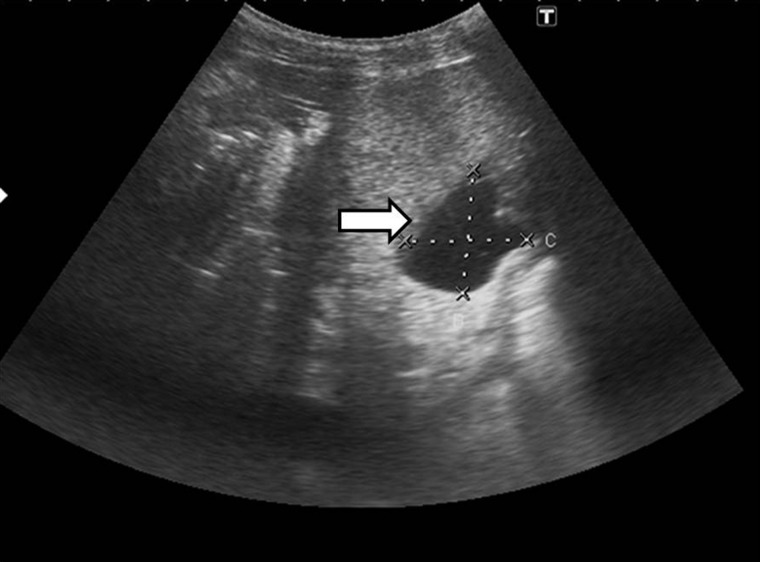
Seroma. Ultrasound image showing collection in the perihepatic location (arrow).

**Figure 6.**
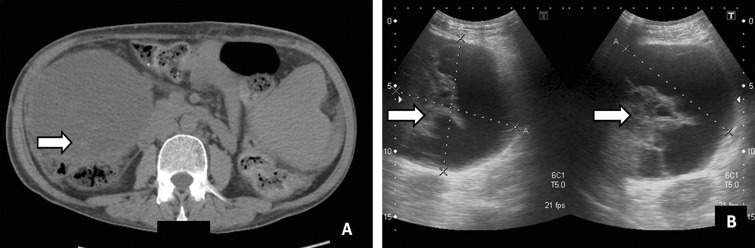
Hematoma. (**A**) Axial non-contrast CT scan image showing collection in the subhepatic location with hyperdense contents within (arrow). (**B**) Ultrasound image showing collection (arrows) with thick internal septations and internal echoes.

Fluid collections such as hematomas, seromas, bilomas, localized ascites and abscesses can impact graft survival. Seromas and hematomas are seen in first few days after transplantation near areas of vascular anastomosis (hepatic hilum, IVC) or the perihepatic/subhepatic space (Figures 5 and 6). On ultrasound, collections may appear completely anechoic, loculated or show fine internal echoes due to fibrin septa or blood components. On multidetector CT, acute hematomas are hyperdense and chronic hematomas show a dependent hyperdense hematocrit level. Magnetic resonance imaging (MRI) shows signal characteristics of fluid or hemorrhage depending upon the nature of collection. Abscesses can occur as a result of bacteremia or superinfection of a pre-existing collection or infarcted/ischemic liver area. On imaging, abscesses show peripheral, thick irregular rim enhancement, intralesional gas and diffusion restriction on diffused-weight (DW)-MRI. The role of imaging is to identify the site and amount of the fluid collection in order to plan interventional procedures if required [9].

## Abdominal complications

**Figure 7.**
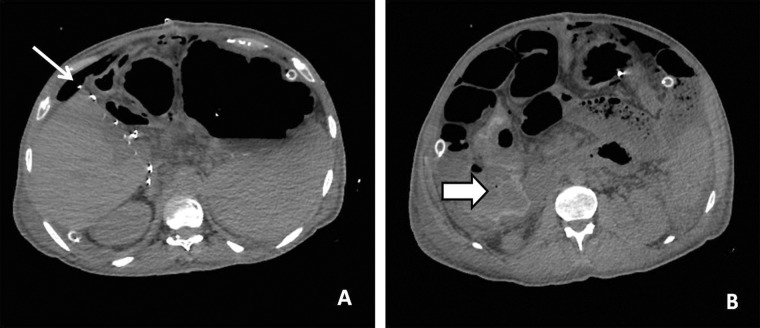
Intestinal perforation. (**A,B**) Axial non-contrast CT scan images showing free extraluminal air in the peritoneal cavity (arrows) and active contrast extravasation in a case of intestinal perforation (arrowhead).

Gastrointestinal (GI) complications post liver transplant include gastrointestinal bleeding, obstruction, internal and external hernias, perforation (Figure 7), pneumatosis intestinalis, mesenteric ischemia, stercoral colitis and malposition of catheters. GI bleeding has been observed in 2.4–25% of recipients. Causes of bleeding in the early postoperative period (within 30 days) are variceal bleeding, peptic ulcer bleeding and bleeding from jejunostomy or hepaticojejunostomy sites. The cause of late GI bleeding includes variceal bleeding due to persistent portal hypertension and mucosal bleeding due to bowel ischemia, infectious enterocolitis, post-transplant lymphoproliferative disorder (PTLD) or graft-versus-host disease [39].

**Figure 8.**
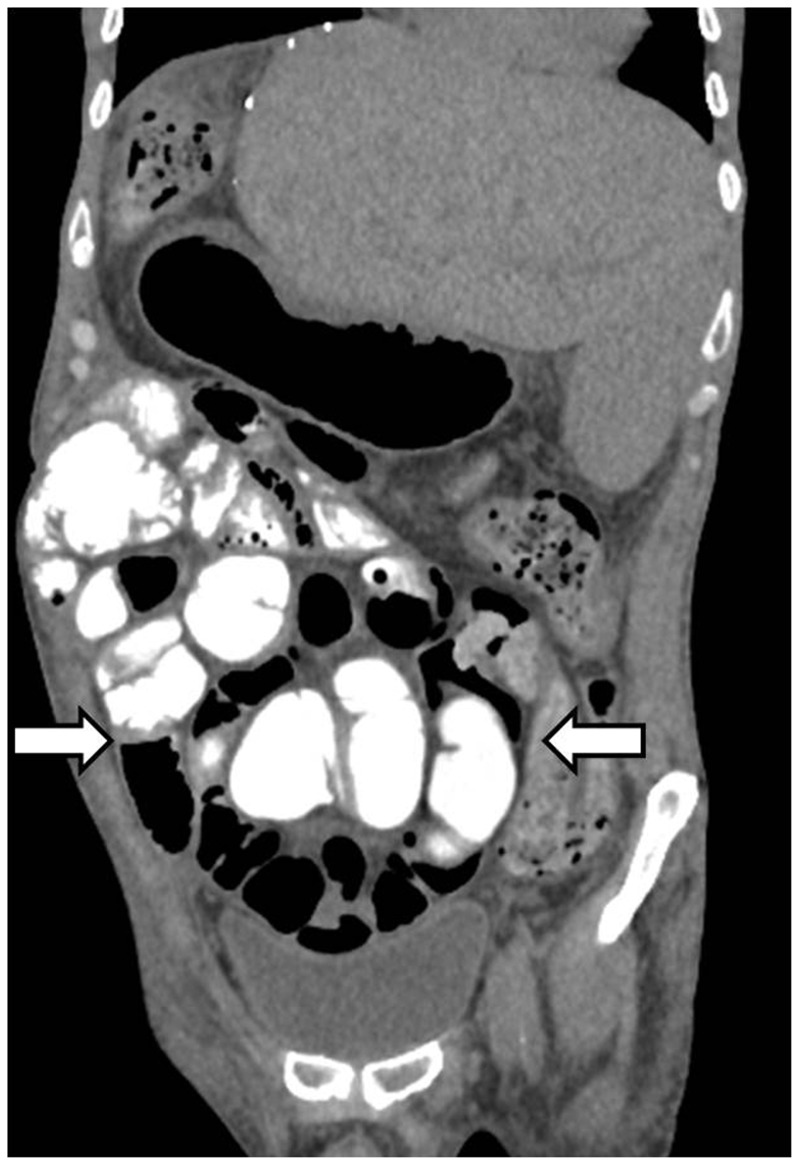
Internal hernia. Coronal oblique reformatted image showing clumped and dilated small bowel loops in a case of internal hernia.

Various causes of obstruction in postoperative patients are gastroparesis, ileus and adhesions. Internal hernias are seen in patients with Roux-en-Y loop hepaticojejunostomy where the herniation occurs through the created mesenteric defect. On CT, a transmesenteric hernia is suggested when there is proximal small bowel dilatation with a transition zone, clustering of small bowel loops and swirling of the mesenteric vessels (Figure 8). Incisional hernia is a frequent complication following liver transplantation, with an incidence rate of 5–17%. Risk factors identified are steroid bolus therapy, low platelet count after transplantation and a bilateral subcostal incision with midline extension [40].

Pneumatosis intestinalis may have an incidental and indolent presentation in 61% of cases after two weeks of surgery. Fulminant pneumatois intestinalis is uncommon and ominous; the findings are small bowel involvement, caliber changes in mesenteric vessels, portomesenteric air embolism, visceral infarction, hemorrhagic ascites and small bowel ileus [41]. Thromboembolism and dissection of the superior mesenteric artery may be rarely encountered, particulartly if there is pre-existing celiac stenosis. Malposition of enteral catheters may also be encountered on imaging.

**Figure 9.**
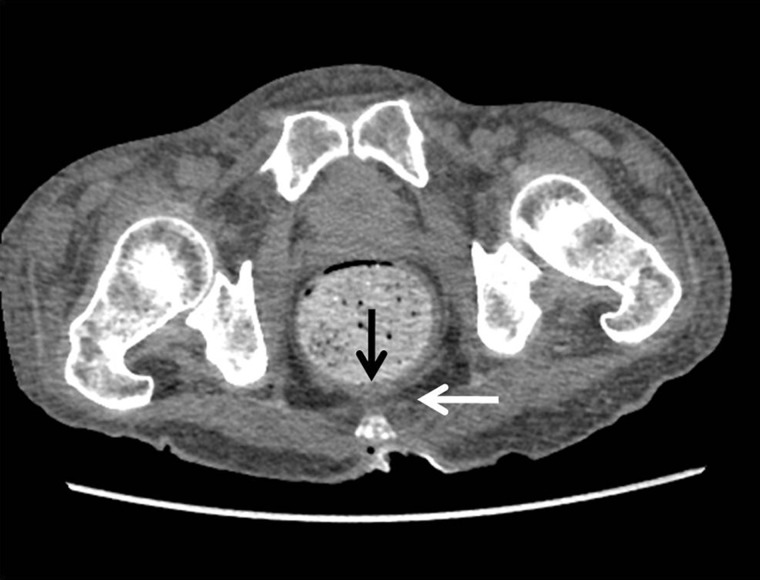
Stercoral colitis. Axial CT image showing dilated colon with impacted fecal material, focal inflammatory wall thickening (black arrow) and fat stranding (white arrow).

Stercoral colitis is an inflammatory colitis due to raised intraluminal pressure from impacted fecal material. On imaging, dilated colon with impacted fecal material, focal thickening of bowel wall, inflammatory wall thickening and fat stranding with extraluminal air may be seen [42] (Figure 9). *Clostridium difficile* colitis (CDC) is also one of the serious complications after liver transplantation. Albright *et al*., in their study of 467 consecutive liver transplant patients, concluded that CDC within one year post transplant was significantly more likely to have a hemorrhagic, biliary or infectious complication, and those who developed CDC within 28 days post transplant had a significantly higher MELD (model for end-stage liver disease) score [43].

## Pulmonary complications

**Figure 10.**
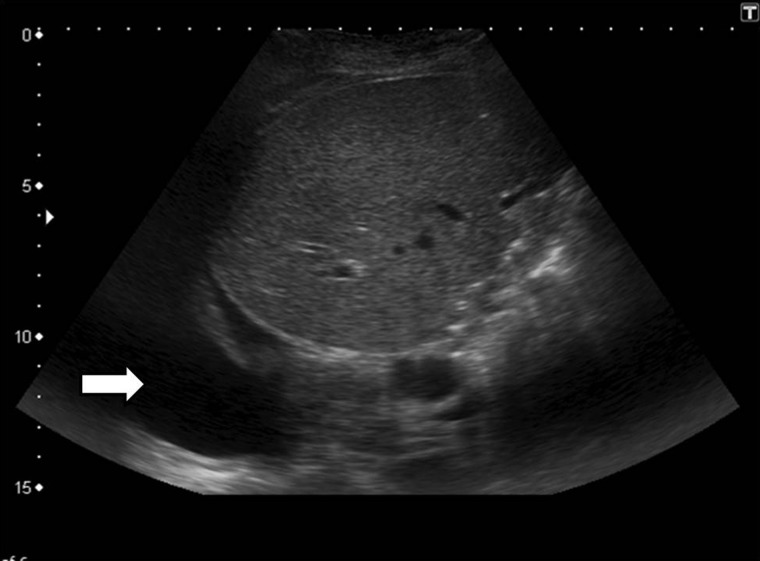
Pleural effusion. Ultrasound image showing right pleural effusion (arrow) with passive atelectasis.

Pleural effusion mainly involves the right side due to disruption of the diaphragmatic lymphatics during hepatectomy (Figure 10). These effusions are usually asymptomatic and self-limiting, and chest tube placement is rarely required. If the effusion increases beyond the first week or remains isolated to the left side, the fluid should be sampled to rule out other causes. Persistent pleural effusions lead to passive atelectasis or predispose to development of pneumonia. Postoperative atelectasis can also be the result of bronchial obstruction due to secretions or defective expulsion mechanism.

**Figure 11.**
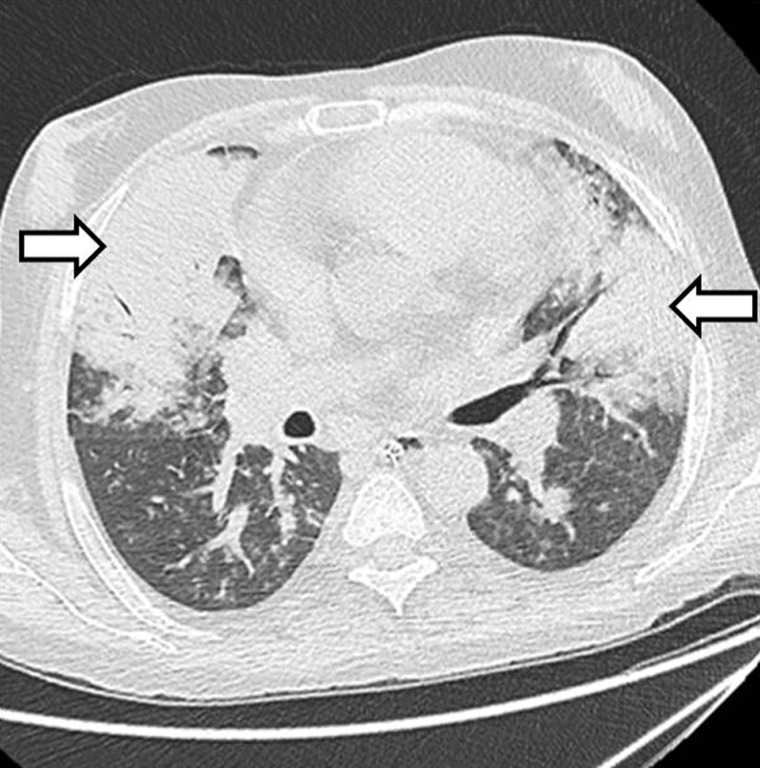
Fungal pneumonia. Axial high resolution CT lung window scan showing *Aspergillus* infection in the form of dense mass-like consolidation (arrows) in bilateral lungs with surrounding areas of ground-glass opacities.

The incidence of post-transplant pneumonia varies from 5–38%. Hospital-acquired pneumonia is usually seen in the first six days of hospitalization. Ventilator-associated pneumonia is defined as pneumonia occurring > 48 hours after patients have been intubated and received mechanical ventilation. Common micro-organisms are gram-negative pathogens such as *Pseudomonas aeruginosa*, *Escherichia coli*, *Klebsiella* species, *Acinetobacter* species and *Staphylococcus aureus* (including methicillin-resistant *Staphylococcus aureus*). Tuberculosis is more frequent in transplant recipients and is mainly observed in locales that are highly endemic. The incidence of cytomegalovirus pneumonitis is 0–9.2% in liver transplant recipients. Aspergillosis is the most common and fatal fungal pathogen in transplant recipients (Figure 11). The most important risk factors for invasive aspergillosis are repeat transplantation and renal failure in liver transplant recipients [44].

Pulmonary edema results when the liver recipient experiences acute-onset, severe left ventricular dysfunction or acute fluid overload in the case of renal impairment [45, 46]. On chest radiograph, pulmonary edema is diagnosed by the presence of cardiomegaly, pleural effusion, bat-wing opacities, peribronchial cuffing and septal lines. Risk factors for acute respiratory distress syndrome are crystalloid infusion overload, massive transfusion of blood or blood products, prolonged operating times, bleeding during surgery and severe ischemia-reperfusion syndrome. According to the American-European Consensus Conference, acute respiratory distress syndrome is characterized by the following criteria: lung injury of acute onset, bilateral opacities on chest imaging not explained by other pulmonary pathology, respiratory failure not explained by heart failure or volume overload and decreased arterial PaO_2_:FiO_2_ ratio [47].

Other pulmonary complications include transfusion-related acute lung injury, right diaphragmatic paralysis and elevation due to phrenic nerve injury, alveolar hemorrhage and malposition of tubes and catheters.

## Neurological complications

**Figure 12.**
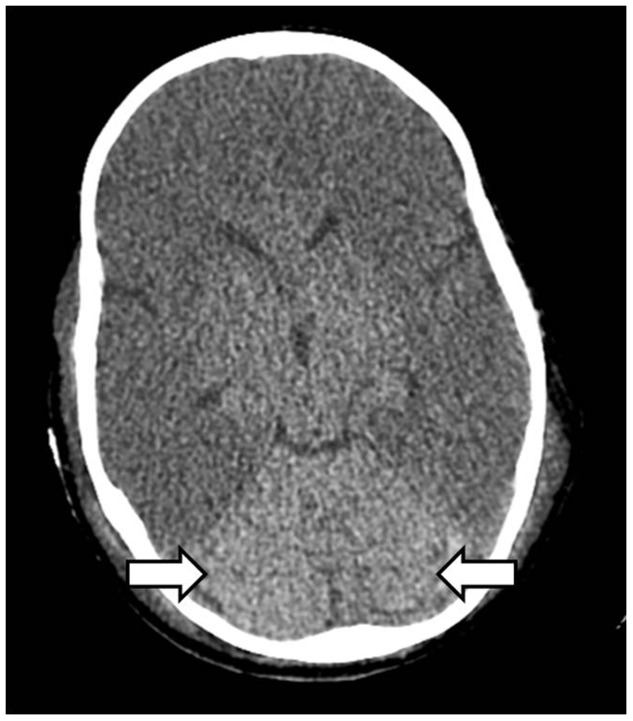
Cerebral edema. Axial non-contrast CT head showing diffuse hypodense cerebral parenchyma with loss of grey-white matter differentiation and classical white cerebellum sign (arrows).

**Figure 13.**
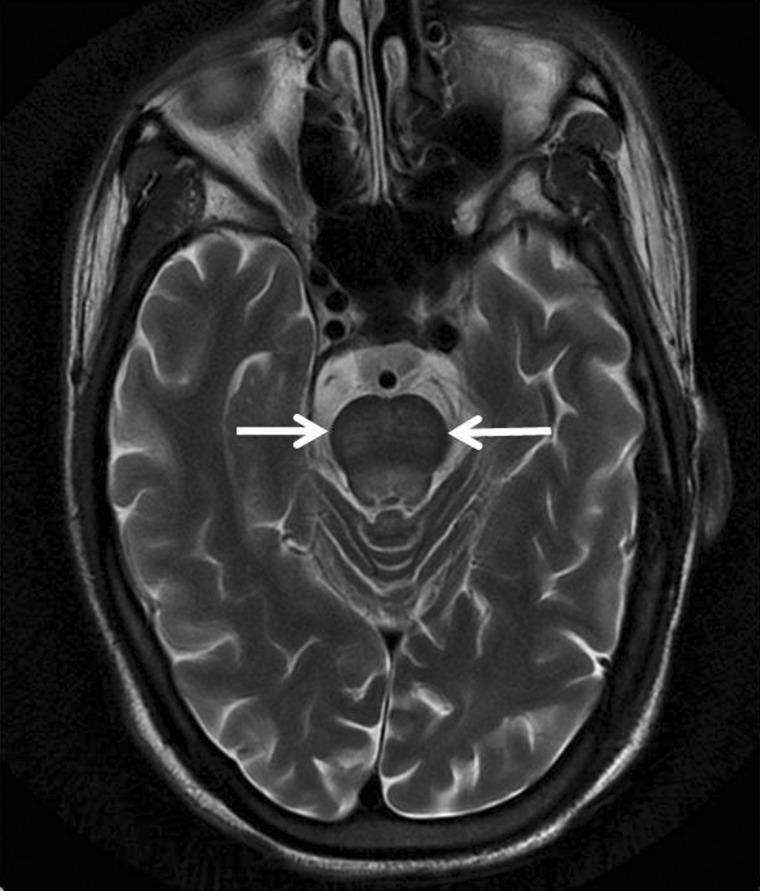
Central pontine myelinolysis. Axial T2-weighted MR brain showing central hyperintensity in pons in a case of pontine myelinolysis.

Various neurological complications after liver transplant are post-transplant encephalopathy, hepatic encephalopathy, cerebral edema (Figure 12), opportunistic central nervous system (CNS) infections due to chronic immunosuppression, central pontine (Figure 13) and extrapontine myelinolysis in the first 48 hours due to rapid correction of hyponatremia, acquired hepatocerebral degeneration, seizures due to focal brain lesions, CNS infections or even posterior reversible encephalopathy syndrome. Cerebrovascular complications such as ischemic strokes and intracranial hemorrhage are rare after liver transplantation, with a reported prevalence of 2–4% in transplant recipients, particularly in older recipients and patients with pre-transplant diabetes. Liver failure resulting from different causes may also manifest with various neurologic symptoms including hepatic encephalopathy, Parkinsonism, asterixis, tremor and hepatic neuropathy. Neurological manifestations may also be specific to causes of liver failure such as Wilson’s disease, alcoholic cirrhosis, hepatitis C virus infection and primary biliary cirrhosis. Immunosuppressive neurotoxicity, progressive multifocal leukoencephalopathy and post-transplant lymphoproliferative disorder may be also seen [48].

## Neoplasms

Neoplasms occurring after liver transplant are either recurrent malignancy, metastatic disease from a separate primary malignancy or PTLD. Due to immunosuppressive therapy, transplanted patients are at higher risk for developing *de novo* malignancies, skin malignancies, Kaposi’s sarcoma, cervical cancer and breast cancer, while patients with inflammatory bowel disease and primary sclerosing cholangitis have an increased risk for developing colorectal cancer. The incidence of PTLD ranges from 1.1–8.4% and most often manifests within the first year after transplantation. PTLD is more frequently extranodal in nature, with intra-abdominal and extra-hepatic disease being common in liver recipients. On imaging, a varied spectrum (involving almost any organ) may be seen that ranges from benign polyclonal lymphadenopathy and poorly defined soft tissue mass encasing or narrowing of the hilar structures to malignant monoclonal lymphoma. The majority of PTLDs consist of B-lymphocyte proliferations activated by Epstein-Barr virus infection [9, 36].

## Rejection

Rejection is the most common cause of graft failure, and imaging has a limited role in diagnosis. Liver biopsy is the gold standard. It is either acute cellular rejection or chronic ductopenic rejection. Acute cellular rejection typically occurs within the first three weeks after transplantation, and chronic ductopenic rejection usually occurs six weeks to six months after transplant [36].

## Conclusion

A varied spectrum of complications may be observed in the post-transplant period. Ultrasound is an economical first method of investigation for vascular and biliary complications and postoperative fluid collections. CT and MRI are complementary imaging modalities. Gadolinium ethoxybenzyl diethylenetriamine penta-acetic acid (Gd-EOB-DTPA)-enhanced MRCP can detect biliary leaks by demonstrating extravasation of contrast material into fluid collections. An understanding of system-wide complications and the imaging modality to be used in a particular complication aids in timely diagnosis and proper management of these patients. This article describes and illustrates the spectrum of imaging appearances of complications associated with liver transplantation.

*Conflict of interest statement*: none declared.
